# Are we reluctant to share our experiences through E-mail and still love postal survey?

**DOI:** 10.4103/0019-5049.60506

**Published:** 2010

**Authors:** Ashok Jadon

**Affiliations:** Tata Motors Hospital, Jamshedpur-831 001, India

Sir,

All of us know that air is a preferred choice of medium during epidural localisation although evidences support the superiority of saline, and also many of us must be practicing epidural blood patch (EBP) for post dural puncture headache (PDPH).

To know the Indian perspective regarding use of saline for epidural localization and use of blood patch in PDPH, I conducted an email-survey using two popular internet sites (used by many of our colleagues) and personal e-mail list of anaesthesia colleagues. A questionnaire was sent with a request to share experiences and opinions about the use of saline for epidural localisation and epidural blood patch for PDPH. The response was very poor - only 10 responses to 100 e-mails (representing only 15 doctors). I really don't know the real cause of this poor response. It could be due to non-importance of these issues as far as modern anaesthesia practices are concerned, or reluctance to share our experiences through e-mail. To know the probable answer of this poor response, literature was searched.

Today, medical practice is evidence-based and, medical audit and surveys helps in data collection to make opinions, decisions and guidelines. In the past when technology was not so advanced and internet facilities were not available, surveys were done either collecting data on data sheets by contacting individual basis or by sending postal quarries (postal survey). Now, due to advancement in technology (patient electronic record) and internet, surveys can be conducted either through direct patients' data retrieval or sending e-mails to participants (patients or care takers) and also electronic methods are proved superior to conventional methods like manually filling forms and postal surveys.[1–3]

Results of recently published meta-analyses of 39 studies within the last 10 years, comparing Web and mail survey modes, showed that college respondents appear to be more responsive to Web surveys, while some other respondents (e.g. medical doctors, school teachers, and general consumers) appear to prefer traditional postal mail surveys.[4]

Many more prospective controlled studies are required before reaching any conclusion regarding better choice for medical surveys. However, this observation highlights the fact that doctors prefer postal survey over e-mail surveys and, therefore, for better response rate we should use postal surveys.
